# Fighting the Antibiotic Crisis: Flavonoids as Promising Antibacterial Drugs Against *Helicobacter pylori* Infection

**DOI:** 10.3389/fcimb.2021.709749

**Published:** 2021-07-20

**Authors:** Andrés González, Javier Casado, Ángel Lanas

**Affiliations:** ^1^ Group of Translational Research in Digestive Diseases, Institute for Health Research Aragón (IIS Aragón), Zaragoza, Spain; ^2^ Department of Medicine, Psychiatry and Dermatology, University of Zaragoza, Zaragoza, Spain; ^3^ Biomedical Research Networking Center in Hepatic and Digestive Diseases (CIBERehd), Madrid, Spain; ^4^ Department of Biochemistry and Molecular & Cellular Biology, University of Zaragoza, Zaragoza, Spain; ^5^ Digestive Diseases Service, University Clinic Hospital Lozano Blesa, Zaragoza, Spain

**Keywords:** *Helicobacter pylori*, flavonoids, plant-derived antimicrobials, antibiotic resistance, natural products

## Abstract

Over half of the world’s population is estimated to be infected with *Helicobacter pylori*. Chronic infection with this microbial class I carcinogen is considered the most important risk factor for developing gastric cancer. The increasing antimicrobial resistance to first-line antibiotics mainly causes the failure of current eradication therapies, inducing refractory infections. The alarming increase in multidrug resistance in *H. pylori* isolates worldwide is already beginning to limit the efficacy of existing treatments. Consequently, the World Health Organization (WHO) has included *H. pylori* in its list of “priority pathogens” for which new antibiotics are urgently needed. Novel strategies must be followed to fight this antibiotic crisis, including properly exploiting the proven therapeutic potential of medicinal plants and plant-derived phytochemicals. In this mini-review, we overview the impressive properties of naturally occurring flavonoids as effective antimicrobial agents against *H. pylori*, which support the use of these plant-derived bioactive compounds as promising drug candidates for inclusion in novel and personalized combinatory therapies against *H. pylori* infection.

## Introduction

*Helicobacter pylori* inhabits the gastric mucosa of almost 4.4 billion people worldwide (Hooi et al., 2017). Without effective eradication therapy, infection usually persists lifelong, causing gastric mucosal inflammation, which may gradually progress to peptic ulcer disease, gastric adenocarcinoma, and mucosa-associated lymphoid-tissue (MALT) lymphoma (Kusters et al., 2006; Yamaoka, 2010). Presently, the efficacy of one-week standard triple therapy containing clarithromycin (CLR) and either metronidazole (MTZ) or amoxicillin (AMX) combined with a proton-pump inhibitor (PPI) has dramatically dropped, showing eradication rates as low as 50% to 70% (Fallone et al., 2016). CLR-containing regimens are no longer suitable for unconditional empiric use because of commonly high levels of antimicrobial resistance and inadequate eradication rates, while the efficacy of the other alternative treatments varies greatly, which usually causes refractory infections. Given the rate at which clinically relevant pathogens, such as *H. pylori*, are acquiring multidrug resistance, the feared possibility that we cannot effectively treat these human bacterial infections is becoming a reality (Boyanova et al., 2016). In 2017, the World Health Organization (WHO) included *H. pylori* in its first list of antibiotic-resistant “priority pathogens”, a catalogue of 12 families of bacteria that presently pose the greatest threat to human health (Tacconelli et al., 2018). Nowadays, effective novel therapy against *H. pylori* is mandatory to increase eradication rates and minimize both antimicrobial resistance and side effects on normal microbiota.

Long before *H. pylori* infection was recognized as causing chronic gastritis and peptic ulcers in 1982 (Marshall and Warren, 1984), natural products have been used by physicians and healers to combat these illnesses based on empirical knowledge (Yesilada et al., 1997). Today, over 240 plant species have demonstrated anti-*H. pylori* activity (Salehi et al., 2018; Baker, 2020). With the pressing need for novel therapeutic options to face the current antibiotic crisis, the scientific community’s interest in traditional medicine and the use of natural products as sources of novel antibacterial drugs have been reinforced (Cheesman et al., 2017; Anand et al., 2019). In this mini-review, we overview the impressive findings obtained due to various studies that focused on the anti-*H. pylori* properties of flavonoids. We also discuss the promising roles of these natural products as potential drug candidates. Finally, we revise the current strategies to improve the bioavailability and efficacy of these phytochemicals.

## Flavonoids: A Large Family of Naturally Occurring Bioactive Compounds

Flavonoids are polyphenolic, low-molecular-weight bioactive compounds ubiquitous in plants (Buer et al., 2010). The flavonoid family comprises over 9,000 species of molecules, which mostly share a chemical structure based on a fifteen-carbon (C6-C3-C6) skeleton comprising two benzene rings denoted as A and B, linked through a heterocyclic pyran ring referred to as ring C. The C6-C3-C6 skeleton is often hydroxylated in positions 2, 3, 5, 7, 3´, 4´, and 5´. Methyl ethers and acetyl esters of the alcohol groups are frequent, although a plethora of other derivative groups, including different alkyls, isoprenoids, and carboxylic groups, also contribute to the vast diversity of these compounds (Kumar and Pandey, 2013). Based on the oxidation state of the central pyran ring, its degree of hydroxylation, and the connection position of benzene ring B, flavonoids could be divided into seven major classes: flavones, flavonols, flavanones, flavanonols, flavanols (also known as flavan-3-ols), anthocyanidins, and isoflavones (**Table 1**).

**Table 1:** Flavonoid classes and their major natural sources.

Flavonoid class	Structure backbone	Examples	Major natural sources
Flavones	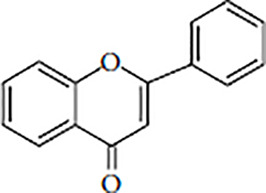	Apigenin Chrysin Luteolin Tangeritin	Celery, parsley, red peppers, chamomile, mint, ginkgo biloba
Flavonols	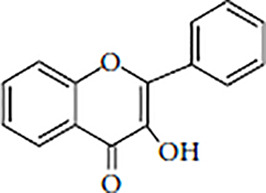	Kaempferol Quercetin Myricetin Fisetin	Onions, kale, lettuce, tomatoes, apples, grapes, berries, tea, red wine
Flavanones	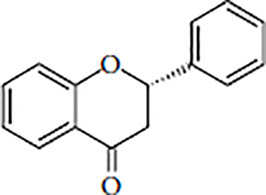	Hesperetin Naringenin Eriodictyol Butin	Citrus fruits, grapes, rice
Flavanonols	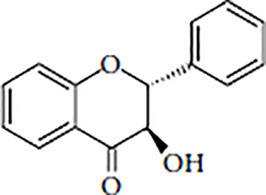	Taxifolin Aromadedrin Engeletin Astilbin	Citrus fruits, tea, rice
Flavanols (Flavan-3-ols)	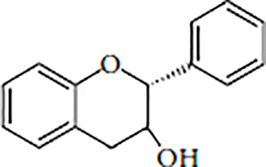	Catechin Epicatechin Gallocatechin Proanthocyanidins	Tea, cocoa, bananas, apples, blueberries, peaches, pears, grapes, red wine
Anthocyanidins	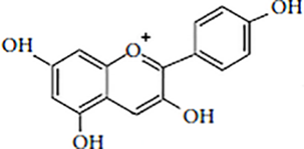	Malvidin Cyanidin Delphinidin Petunidin	Berries, black currants, red grapes, merlot grapes
Isoflavones	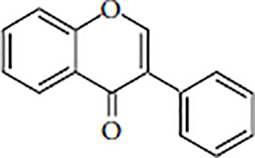	Daidzein Genistein Glycitein Formononetin	Legumes

Flavonoids are synthesized as secondary metabolites by all plant organs. These natural polyphenols are critical in plants’ interaction with other organisms, such as microorganisms, animals, and other plants, but they also participate in responding to different abiotic stresses, including UV radiation, extreme temperatures, heavy metals, and droughts (Mierziak et al., 2014). A major role of flavonoids in plants is their function as a second line of defense against oxidative stress. Flavonoids can inhibit the generation of reactive oxygen species (ROS) by several mechanisms (Kumar and Pandey, 2013), but they also quench ROS once they are produced. Some environmental stresses, such as drought, salinity, extreme temperatures, and nutrient scarcity, may significantly reduce the activity of ROS-detoxifying enzymes in chloroplasts. As an adaptive response, plants upregulate the biosynthesis of ROS-scavenging flavonoids. These polyphenols will not only absorb the most energetic solar wavelengths (i.e., UV-B and UV-A) but will also scavenge free metal ions, peroxyl, superoxide, and peroxynitrite radicals, thereby avoiding lipid peroxidation and oxidative damage to other biomolecules (Kumar and Pandey, 2013; Del Valle et al., 2020).

## Mechanisms of Antimicrobial Action—A Close Relationship Between Structure and Activity

As with other phytochemicals, the antimicrobial activity of flavonoids appears multifactorial while acting against different molecular targets in the pathogen instead of having one specific action site. However, the presence of certain structural features in the flavonoid molecule enhances its pharmacological effects, reinforcing one or another action mechanism, suggesting a relationship between the flavonoid structure and its antiviral and/or antimicrobial activities (Cushnie and Lamb, 2005; Kumar and Pandey, 2013; Wang et al., 2018; Farhadi et al., 2019; Górniak et al., 2019). Thus, while a greater abundance in hydroxyl groups increases the antioxidant effects of flavonoids due to a higher number of functional sites for scavenging free radicals and chelating metal ions, this high degree of hydroxylation diminishes simultaneously with flavonoid lipophilicity, thereby limiting the influx of these molecules across the pathogen cell membranes. Hence, lipophilic flavonoids, such as herperetin, naringenin, sophoraflavanone G, and catechins with gallate groups, could penetrate the lipid bilayer membrane up to the zone under phosphate groups and laterally diffuse into the bilayer plane, causing alterations in membrane fluidity and permeability (Tsuchiya and Iinuma, 2000; Tarahovsky et al., 2014). Other flavonoids such as quercetin cause a decrease in the proton-motive force impairing the production of adenosine triphosphate (ATP), while apigenin and morin induce destabilization of the membrane structure by the disordering and disorientation of membrane lipids (Górniak et al., 2019).

Also, hydrophilic flavonoids could interact at the membrane surface and/or in the cytosol with proteins involved in different essential functions, including adhesins, cell envelope transporters, transcriptional regulators, enzymes, and toxins, inactivating these biomolecules by forming flavonoid-protein complexes through hydrophobic interactions, hydrogen, and/or covalent bonds (Kumar and Pandey, 2013; Górniak et al., 2019). Notably, little difference in the molecular structure of two flavonoids could be responsible for different effectivities in their capability to inhibit the biological activity of the same protein target. For instance, both apigenin and quercetin inhibited the function of D-alanine:D-alanine ligase (Ddl) using the same inhibition mechanism as competing with the substrate ATP (Wu D. et al., 2008). Although these two flavonoids only differ in the two additional hydroxyl groups that quercetin possesses at positions 3 and 3´, this little difference induces a substantial increase in the affinity of quercetin by the active site of the enzyme, resulting in at least a three-fold increase in its inhibitory activity against Ddl regarding apigenin. However, quercetin exhibited lower antibacterial activity than apigenin, which could be a consequence of poorer transport across cell membranes due to lower lipophilicity (Wu D. et al., 2008).

## Natural Flavonoids Against *Helicobacter pylori* Infection

Although many published studies have described the therapeutic potential of different plant extracts and other flavonoid-rich natural products (Ankolekar et al., 2011; Njume et al., 2011; Takeuchi et al., 2014; Wang, 2014; Boyanova et al., 2015; Salehi et al., 2018; Baker, 2020; Mendonca et al., 2020), we focused this mini-review on the advances in the knowledge of the antimicrobial activities of natural purified flavonoids against *H. pylori* (**Table 2**). Notably, several flavonoids have exhibited potent antimicrobial activities (MIC ≤ 8 µg/mL) against *H. pylori*. These *in vitro* antimicrobial potencies are comparable with those exhibited by some conventional antibiotics traditionally used in anti-*H. pylori* therapies, such as metronidazole, against sensitive strains (Loo et al., 1997). Although the anti-*H. pylori* activity exhibited by these flavonoids is probably multifactorial, an increasing number of studies have successfully identified specific molecular targets of these bioactive compounds in *H. pylori*, unravelling both antimicrobial and antivirulence mechanisms. Thus, several bactericidal flavonoids noticeably inhibited the essential function of HsrA (González et al., 2019), an OmpR-like orphan response regulator (Lee et al., 2006), which acts as a global homeostatic regulator synchronizing metabolic functions and virulence with the availability of nutrients and cell division, also mediating the response to oxidative stress (Olekhnovich et al., 2013; Olekhnovich et al., 2014; Pelliciari et al., 2017). Isothermal titration calorimetry studies indicated that chrysin, apigenin, kaempferol, and hesperetin bind to HsrA with dissociation constants in the micromolar range, showing a 1:1 stoichiometry. Molecular docking analyses suggest that interactions between these flavonoids and HsrA preferably occur by the C-terminal effector domain of the response regulator, thereby blocking its interaction with DNA (González et al., 2019). Notably, apigenin, kaempferol, and hesperetin also affected other recognized molecular targets in *H. pylori*, including enzymes (Wu D. et al., 2008; Zhang et al., 2008), secretion systems (Yeon et al., 2019), and cell membranes (Moon et al., 2013).

**Table 2:** Natural flavonoids with antimicrobial activities against *H. pylori*.

Class	Flavonoid	MIC (µg/mL)	Target^1^	Additive or synergy^2^	References
Flavones	Chrysin	4	HsrA	CLR, MTZ	(González et al., 2019)
	Apigenin	8	HsrA, FabZ, Ddl		(Isobe et al., 2006; Wu D. et al., 2008; Zhang et al., 2008; Wang and Huang, 2013; Kuo et al., 2014; González et al., 2019)
	Luteolin	32	HsrA, NAT, Urease		(Chung et al., 2001; Isobe et al., 2006; Moon et al., 2013; González et al., 2019; Tran Trung et al., 2020)
	Nobiletin		PDF		(Rajesh et al., 2013; Ouyang et al., 2020)
	Diosmin		Urease		(Kataria and Khatkar, 2019a)
	Baicalin	450	Urease, VacA	AMX, TET	(Wu J. et al., 2008; Huang et al., 2015; Yu et al., 2015; Chen et al., 2018)
	Baicalein	33.7	VacA		(Matsumoto et al., 2008; Chen et al., 2018)
	Isoorientin				(Yuan et al., 2018)
	Acacetin	62.5			(Gomez-Chang et al., 2018)
	Diosmetin				(Gomez-Chang et al., 2018)
	Scutellarin		Urease		(Yu et al., 2015; Chledzik et al., 2018)
	Oroxindin	50	Urease	AMX	(Fong et al., 2019)
	Galangin				(Skiba et al., 2016)
	Sudachitin				(Nakagawa et al., 2006)
	Cirsimaritin	6.3			(Isobe et al., 2006)
	Cirsilineol	3.2			(Isobe et al., 2006)
	Sinensetin	25			(Isobe et al., 2006)
	Eupatorin	12.5			(Isobe et al., 2006)
	Pedalitin	25			(Isobe et al., 2006)
Flavonols	Quercetin	64	HsrA, FabZ, Ddl, Urease		(Shin et al., 2005; Gonzalez-Segovia et al., 2008; Wu D. et al., 2008; Zhang et al., 2008; Martini et al., 2009; Brown et al., 2011; Xiao et al., 2012; Brown and Jiang, 2013; Moon et al., 2013; Macomber et al., 2015; Haghi et al., 2017; Zhang et al., 2017; González et al., 2019)
	Kaempferol	8	HsrA, VacA, T4SS, T5SS		(Martini et al., 2009; Bisignano et al., 2013; Moon et al., 2013; González et al., 2019; Yeon et al., 2019)
	Myricetin	128	HsrA, Urease, spiral-to-coccoid transition	CLR, MTZ, LVX, TET, AMX	(González et al., 2019; Tran Trung et al., 2020; Krzyzek et al., 2021)
	Morin		Urease		(Kataria and Khatkar, 2019b)
	Isorhamnetin	3.9			(Ustun et al., 2006; Bisignano et al., 2013)
Flavanones	Hesperetin	4	HsrA, cell membrane	MTZ, CLR	(Lee et al., 2012; Moon et al., 2013; González et al., 2019)
	Naringenin	128	Urease, biofilm		(Shin et al., 2005; Bisignano et al., 2013; Moon et al., 2013; Tran Trung et al., 2020)
	Sakuranetin	25	FabZ		(Zhang et al., 2008; Stompor, 2020)
Flavanols	Catechin				(Bisignano et al., 2013; Silvan et al., 2020)
	Epicatechin	128			(Bisignano et al., 2013)
	Epigallocatechin		Urease		(Macomber et al., 2015)
	Proanthocyanidins		Urease		(Wittschier et al., 2007; Hribova et al., 2014; Pastene et al., 2014; Silvan et al., 2020)
Anthocyanidins	Cyanidin		SecA, T5SS		(Kim et al., 2012; Kim et al., 2014; Kim et al., 2018)
Isoflavones	Daidzein		Urease		(Ndemangou et al., 2013)

^1^FabZ, β-hydroxyacyl-acyl carrier-protein dehydratase; Ddl, D-Alanine:D-alanine ligase; NAT, N-acetyltransferase; PDF, Peptide deformylase. T4SS and T5SS: bacterial type IV and type V secretion systems.

^2^Additive or synergistic effect according to the checkerboard assay (White et al., 1996). CLR, clarithromycin; MTZ, metronidazole; TET, tetracycline; AMX, amoxicillin; LVX, levofloxacin.

Many naturally occurring flavonoids exhibit anti-urease activity (**Table 2**). Molecular docking studies and structure–activity relationship analyses proved that 3-OH, 5-OH, and 3′,4′-dihydroxyl groups of quercetin generate hydrogen bonds with amino acid residues of *H. pylori* urease, which appear essential for the inhibitory activity exerted by this flavonoid. Removing or substituting any of these functional hydroxyl groups from the quercetin structure significantly decreases its urease inhibitory activity (Xiao et al., 2012). The critical impact of OH groups on the affinity and half maximal inhibitory concentration (IC50) of flavonoids against *H. pylori* enzymes has also been demonstrated in other studies (Wu D. et al., 2008; Yu et al., 2015). Flavonoids’ value as effective anti-*H. pylori* therapeutic drugs is not only supported by their proven bactericidal effect but also due to their antivirulence actions, which in many cases reduce damage to the host and alleviate associated diseases. Some *H. pylori* virulence factors, including cytotoxin-associated gene A (CagA) and vacuolating cytotoxin A (VacA), are critical in the inflammation process associated with infection with this pathogen. Cytotoxin CagA (Ansari and Yamaoka, 2020), encoded by the *cag* pathogenicity island, is translocated to host cells *via* the type IV secretion system (T4SS), a sophisticated transmembrane protein complex that directly injects the toxin into gastric epithelial cells (Backert et al., 2017). Once into the target cells, CagA activates NF-κB, a master regulator of immune and inflammatory responses that modulates the gene expression of pro-inflammatory cytokines, such as IL-8, TNF-α, and IL-1β (Lamb and Chen, 2013). Additionally, the cytotoxin VacA (Palframan et al., 2012), which is secreted from *H. pylori via* the type V secretion system (T5SS), acts on the host cell, inducing vacuolation and apoptosis, and also increases IL-8 production by activating the p38 MAPK *via* intracellular Ca^2+^ release, thereby activating the transcription factors, ATF-2, CREB, and NF-κB (Hisatsune et al., 2008). Several flavonoids, including apigenin (Wang and Huang, 2013), kaempferol (Yeon et al., 2019), quercetin (Gonzalez-Segovia et al., 2008; Zhang et al., 2017), nobiletin (Ouyang et al., 2020), baicalin, baicalein (Chen et al., 2018), galangin (Skiba et al., 2016), and genistein (Siriviriyakul et al., 2020) have shown protection against gastric inflammation associated with *H. pylori* infection by reducing pro-inflammatory cytokine expression. Thus, kaempferol decreased the mRNA levels of IL-8, TNF-α, and IL-1β in gastric adenocarcinoma cells infected with *H. pylori* by inhibiting *vacA* expression and suppressing CagA and VacA translocation to target cells by inhibiting the expression of several T4SS and T5SS components (Yeon et al., 2019). Quercetin significantly reduced *in vivo* gastric inflammation in *H. pylori*-infected mice by reducing IL-8 secretion and downregulating the p38 MAPK signaling pathway (Zhang et al., 2017). Apigenin decreased the levels of IL-8 by inhibiting the activation of NF-κB (Wang and Huang, 2013). Adding to their anti-inflammatory actions, several flavonoids have been demonstrated to protect against vacuolation, apoptosis, and lipid peroxidation induced by *H. pylori* in gastric mucosa (Shin et al., 2005; Gonzalez-Segovia et al., 2008; Zhang et al., 2017).

A further benefit of flavonoids as antimicrobials against *H. pylori* infections lies in their capacity for synergism combined with anti-*H. pylori* first-line antibiotics, including CLR, MTZ, or AMX (**Table 2**). Notably, chrysin induced an eight-fold decrease in the MIC value of CLR (FIC = 0.125), and caused a 16-fold decrease in the MIC value of MTZ (FIC = 0.0625) (González et al., 2019). Likewise, hesperetin led to a 4-fold increase in the inhibitory activity of MTZ and a two-fold increase in the CLR anti-*H. pylori in vitro* activity (González et al., 2019). Although the molecular mechanisms by which flavonoids enhance the antimicrobial activities of conventional antibiotics remain poorly understood, some experimental evidence unravels putative synergistic interactions. Thus, the increased inhibitory activities of AMX and TET in multidrug-resistant strains of *H. pylori* after baicalin action appeared to be associated with a decrease in the expression of the efflux pump gene *hefA* (Huang et al., 2015). *hefA* encodes a TolC-like outer membrane channel tunnel protein that interacts with different inner-membrane translocases to form efflux systems involved in drug resistance (Liu et al., 2008). Myricetin strongly inhibited the expression of genes involved in the morphological transition of *H. pylori* from spiral to coccoid forms, thereby avoiding the increase in antimicrobial resistance associated with cell shape transformation, which has been observed in this pathogen. Consequently, myricetin induced a 4–16-fold reduction in the MIC values of CLR, MTZ, LVX, TET, and AMX (Krzyzek et al., 2021). In addition, several authors suggest that the proven damage triggered by certain flavonoids in the cytoplasmic membrane and/or cell wall could enhance the susceptibility of bacterial pathogens to the action of antibiotics (Amin et al., 2015; Sanhueza et al., 2017).

## Challenges and Perspectives in Using Flavonoids as Antimicrobials

Low solubility, poor permeability, relative chemical instability, rapid release, and susceptibility to environmental influences, but mainly low bioavailability, contribute to the fact that the *in vivo* exposure levels of flavonoids are usually inconsistent and much below the effective concentrations observed in the *in vitro* studies. Most flavonoids undergo sulfation, methylation, and glucuronidation in the small intestine and liver due to phase 2 metabolism reactions, resulting in more hydrophilic conjugated metabolites, which show reduced bioactivity compared to parent compounds (D’Archivio et al., 2010; Thilakarathna and Rupasinghe, 2013; Hu et al., 2017; Yang et al., 2020).

In recent years, many advanced nanoparticles have been developed not only to improve polyphenol bioavailability but also to control/target their release. Liposomes, phospholipid complexes, niosomes, protein-based nanoparticles, micelles, emulsions, and metal nanoparticles have been demonstrated to significantly increase bioavailability and improve the pharmacokinetics of polyphenols, becoming promising options for flavonoid delivery systems (Chen et al., 2020; Riaz et al., 2020; Yang et al., 2020). Another approach to improving the bioavailability of flavonoids is the rational modification of their molecular structures to bypass phase 2 metabolism during absorption. Some chemical modifications of bioactive molecules generate inactive forms, known as prodrugs, which can usually be transformed into their active forms by a single-step reaction after ingestion. Designing different synthetic prodrugs of polyphenols by capping phenolic hydroxyls with different protecting groups could increase bioavailability and reinforce the therapeutic properties of these bioactive compounds (Biasutto and Zoratti, 2014; Biasutto et al., 2017). Finally, phyto-phospholipid complexes have emerged as a promising strategy for enhancing the bioavailability of bioactive polyphenols. Phytosomes are obtained by complexing phytochemicals with phospholipids at defined molar ratios and under certain conditions. The resulting complexes are more readily absorbed and exhibit higher bioavailability than free bioactive compounds (Lu et al., 2019; Riva et al., 2019).

## Discussion

Antibiotic resistance is among the greatest threats to global health in this century. An impressive accumulation of antibiotic resistance genes by clinically relevant bacterial pathogens, jointly with the present slowdown in developing new antibiotics, is inducing untreatable infections worldwide. *H. pylori* is a carcinogenic bacterium that infects over half of the global population, causing chronic progressive gastric inflammation and various diseases, including gastric and duodenal ulcers and gastric cancer. This malignancy constitutes the fifth most common cancer and the third leading cause of cancer-related mortality globally, representing 9% of all cancer-related deaths worldwide. Although the eradication of *H. pylori* infection has been proven to significantly reduce gastric cancer incidence, the efficacy of current eradication therapies has dramatically decreased, mainly because of an increasing development of antibiotic resistance. In this context, different R&D strategies must be urgently considered to fast-track novel, effective therapeutic options against *H. pylori* infection. Hence, validating novel therapeutic targets, repurposing the existing drugs, using synergistic combinatory therapies, and properly exploiting the proven therapeutic potential of medicinal plants and other natural products could accelerate the delivery of new antimicrobials and the design of novel and personalized treatments against *H. pylori* refractory infections.

Since most of the currently prescribed antibiotics have been obtained from microbial sources or derivatives thereof, using bioactive phytochemicals to develop new antibiotics could overcome the circulating resistome and slowdown the emergence and dissemination of novel antibiotic resistance mechanisms. Among these naturally occurring substances, flavonoids stand out for their multiple and remarkable beneficial effects on human health. Several flavonoids have revealed potent antimicrobial activities against *H. pylori*, in some cases, at the level achieved by first-line antibiotics, such as metronidazole. In addition, some of these polyphenolic molecules exhibited synergistic effects combined with conventional antibiotics, thereby reverting antibiotic resistant phenotypes. Furthermore, the antivirulence actions of these compounds against *H. pylori* contribute to protecting against gastric inflammation, vacuolation, apoptosis, and lipid peroxidation, reducing the damage exerted by the pathogen to the host cells, and decreasing the progression of associated diseases. Although low bioavailability contributes to decreasing the *in vivo* effectivity of natural flavonoids, the current development of novel delivery systems, such as prodrugs, phytosomes, and several nanotechnology approaches, enables the inclusion of flavonoids as novel therapeutic tools against *H. pylori* infection.

## Author Contributions

AG, JC, and ÁL wrote the review. All authors contributed to the article and approved the submitted version.

## Funding

This work has been supported by the Government of Aragon, Spain (B25_17R) and University of Zaragoza (2018/0420).

## Conflict of Interest

The authors declare that the research was conducted in the absence of any commercial or financial relationships that could be construed as a potential conflict of interest.
